# Spatial Segregation and Aggregation of Ectomycorrhizal and Root-Endophytic Fungi in the Seedlings of Two *Quercus* Species

**DOI:** 10.1371/journal.pone.0096363

**Published:** 2014-05-06

**Authors:** Satoshi Yamamoto, Hirotoshi Sato, Akifumi S. Tanabe, Amane Hidaka, Kohmei Kadowaki, Hirokazu Toju

**Affiliations:** 1 Graduate School of Human and Environmental Studies, Kyoto University, Kyoto, Japan; 2 National Research Institute of Fisheries Science, Fisheries Research Agency, Yokohama, Japan; 3 Network center of Forest and Grassland Survey in Monitoring Sites 1000 Project, Japan Wildlife Research Center, c/o Filed Science Center for Northern Biosphere, Hokkaido University, Tomakomai, Japan; Jyväskylä University, Finland

## Abstract

Diverse clades of mycorrhizal and endophytic fungi are potentially involved in competitive or facilitative interactions within host-plant roots. We investigated the potential consequences of these ecological interactions on the assembly process of root-associated fungi by examining the co-occurrence of pairs of fungi in host-plant individuals. Based on massively-parallel pyrosequencing, we analyzed the root-associated fungal community composition for each of the 249 *Quercus serrata* and 188 *Quercus glauca* seedlings sampled in a warm-temperate secondary forest in Japan. Pairs of fungi that co-occurred more or less often than expected by chance were identified based on randomization tests. The pyrosequencing analysis revealed that not only ectomycorrhizal fungi but also endophytic fungi were common in the root-associated fungal community. Intriguingly, specific pairs of these ectomycorrhizal and endophytic fungi showed spatially aggregated patterns, suggesting the existence of facilitative interactions between fungi in different functional groups. Due to the large number of fungal pairs examined, many of the observed aggregated/segregated patterns with very low *P* values (e.g., < 0.005) turned non-significant after the application of a multiple comparison method. However, our overall results imply that the community structures of ectomycorrhizal and endophytic fungi could influence each other through interspecific competitive/facilitative interactions in root. To test the potential of host-plants' control of fungus–fungus ecological interactions in roots, we further examined whether the aggregated/segregated patterns could vary depending on the identity of host plant species. Potentially due to the physiological properties shared between the congeneric host plant species, the sign of hosts' control was not detected in the present study. The pyrosequencing-based randomization analyses shown in this study provide a platform of the high-throughput investigation of fungus–fungus interactions in plant root systems.

## Introduction

In terrestrial ecosystems, various functional groups of root-associated fungi interact with plants [1]. Ectomycorrhizal and arbuscular mycorrhizal fungi are mutualistic partners of more than 80% of terrestrial plant species, enhancing host plant growth and survival by transporting soil nutrients [2]-[5], protecting host plants from pathogens and herbivores [6]–[8], and reducing competition among co-occurring plant individuals/species [9], [10]. Diverse clades of root endophytic fungi, which do not form mycorrhizae, also interact with diverse phylogenetic groups of plants [11], [12]. Some clades of those endophytic fungi are known to enhance the nutritional conditions of host plants [13], [14], whereas many others inhabit plant roots as commensalistic symbionts or parasites [15]. Those different functional groups of root-associated fungi often co-occur in plant roots [16], [17], and understanding the assembly processes of those ecologically and phylogenetically diverse fungi in root systems is one of the major challenges in fungal ecology.

Competitive and facilitative interactions between fungal species are considered to be the major factors responsible for the community organization of fungi in roots [18]–[20]. Fungal species in roots can compete with each other by impeding the colonization of others [21]–[24] or by expelling other species from host roots [25]. These competitive interactions between fungal species in roots, importantly, are expected to result in fine-scale segregated distributions of competing species [26], [27]. Interactions between fungal species can be competitive even between fungi in different functional or phylogenetic groups. In the roots of a *Eucalyptus* plant, for example, ectomycorrhizal fungi prevent arbuscular mycorrhizal fungi from infecting and proliferating in the host [28], [29]. On the other hand, the presence of a fungal species in roots does not always negatively affect the colonization of others. A morphological observation of fungal hyphae in roots revealed that ectomycorrhizal and root-endophytic fungi coexisted in a single root system of a *Pinus* tree, presumably because the two functional groups of fungi occupied different habitats within the roots [30]. Likewise, recent molecular studies have demonstrated the coexistence of ectomycorrhizal and endophytic fungi within roots [31]–[33]. In these cases, interactions between fungal species may be neutral or even facilitative.

Although competitive or facilitative interactions between fungal species can be assessed by inoculation experiments [22], [24], [34], many of the mycorrhizal and endophytic fungi that dominate root-associated fungal communities in natural forests are unculturable. Therefore, such inoculation-based experimental studies are applicable to only a part of fungus–fungus interactions occurring in the wild. An alternative research approach for elucidating the nature of fungal interspecific interactions is to examine the pattern of the presence/absence of fungal species in host individuals and thereby examine the spatial segregation and aggregation (co-occurrence) patterns. Spatial segregation could result from competitive interactions or differences in niches. On the other hand, spatial aggregation could arise from either species sorting [35], in which a pair of fungi with shared habitat requirements come to exist in a particular root, or from facilitative interactions. Therefore, community-wide analyses of the spatial segregation and aggregation of root-associated fungi are expected to reveal the species pairs that have a role in shaping fungal community structures. Indeed, some recent studies on root-associated fungi have examined such segregation/aggregation patterns and inferred the patterns of possible fungus–fungus ecological interactions [27], [36]

While previous studies analyzed the segregation/aggregation of pairs of root-associated fungi regardless of the effects of host plant species [26], [27], [36], such spatial patterns representing fungus–fungus competitive/facilitative interactions are expected to vary depending on host plant species. For example, if plant species with lower photosynthetic rates provide less carbohydrate to their root symbiont communities, it may promote competition for limited carbon resource and cause competitive exclusion between root-associated fungi [37]. Host plants' preference for a particular microenvironment (e.g., soil moisture) may also indirectly affect the relative competitive ability of root-associated fungi that interact with each other in root systems. By comparing segregation/aggregation patterns of root-associated fungi among different host plant species, we can examine such hypothetical plant-mediated processes of fungus–fungus interactions.

By identifying segregated and aggregated distributions of pairs of root-associated fungi on two oak species, we determined the patterns of fungal interspecific interactions and examined the dependence of such fungus–fungus interactions on background plant species. In a temperate forest in Japan, we first analyzed the community composition of root-associated fungi for the seedlings of co-occurring deciduous and evergreen oak species based on the pyrosequencing of fungal internal transcribed spacer (ITS) sequences. We then conducted a randomization test to detect the spatial segregation and aggregation of pairs of root-associated fungi. Furthermore, we examined whether or not the identity of host-plant species was associated with the spatial segregation or aggregation patterns of root-associated fungi.

## Materials and Methods

### Study area and sampling

The seedling samples were collected in a warm-temperate secondary forest on Mt. Yoshida located in Kyoto City, Japan (35.026°N, 135.786°E). No specific permissions were required for the location/activity. We confirmed that the field study did not involve endangered or protected species. The climate of Kyoto City is characterized by humid summers and dry winters: mean temperature and precipitation over the recent 30 years are 26.0 °C and 566.5 mm in summer (from June to August), and 5.6 °C and 164.9 mm in winter (from December to February) [38]. Mt. Yoshida (alt 121 m), which is a small hill with an area of 14.3 ha, is covered mainly by the two oak species *Quercus serrata* and *Q. glauca* (Fagaceae), while *Pinus densiflora* (Pinaceae) and *Ilex pedunculosa* (Aquifoliaceae) co-occur in the canopy layer. The two dominant oak species belong to different subgenus and have different ecological properties: *Q. serrata* is a deciduous species that occurs in the early stages of the secondary succession of temperate forests [39], while *Q. glauca* is an evergreen species whose seedlings occur both sunny and shaded understory of warm-temperate secondary forests [40]. Note that our field research site is not privately owned and sampling seedling in the research area is not banned.

**Figure 1:**
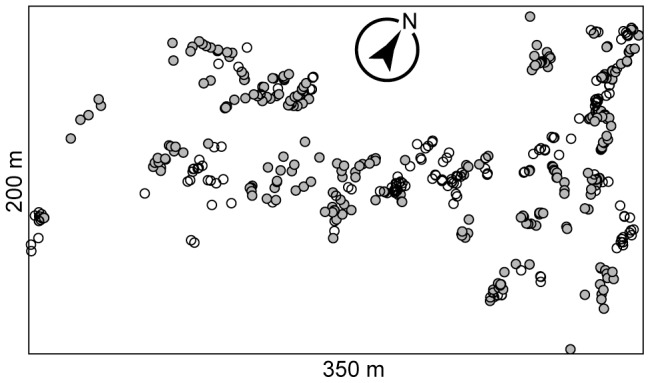
Distribution of the *Quercus* seedlings analyzed on Mt. Yoshida. Circles indicate the sampling locations of the seedlings. In total, 249 *Q. serrata* (gray) and 188 *Q. glauca* (white) seedlings were subjected to the randomization analyses of *C* and *T* scores.

From 20 to 31 May 2011, seedlings of each *Quercus* species were sampled at a minimum interval of 1 m: the number of sampled seedlings was 261 for *Q. serrata* and 199 for *Q. glauca*. The 460 sampling positions were recorded with a GPS device (Germin, GPSMAP 62S; Fig. 1). The size of sampled seedlings was 20–30 cm in height. To sample the seedlings, we dug to a depth of ca. 25 cm, taking great care not to damage the root tips of the seedlings. The amount of the dug soil of each root system was approximately 3,000 cm^3^. The sampled seedlings were individually stored in sealed plastic bags in an ice chest. On the same day of the fieldwork, we randomly collected ten 2-cm fragments of terminal roots per seedling in the laboratory: note that there were seedlings with less than ten 2-cm fragments of terminal roots and hence the number of root fragments collected per seedling ranged from five to ten. The terminal roots were stored in 1.5-ml tubes with 70% ethanol at –20°C until DNA extraction.

### DNA extraction, PCR, and pyrosequencing

To remove soil adhering to roots, 1-mm zirconium balls were introduced into the sample tubes and then the tubes were shaken at 18 Hz for 3 min using TissueLyser II (Qiagen, Hilden, Germany) as detailed elsewhere [33]. After the cleaning, five terminal roots per sample were subjected to fungal DNA extraction. Terminal roots were transferred to a new tube and were pulverized with 4-mm zirconium balls by shaking at 20 Hz for 3 min. DNA extraction was conducted with the cetyltrimethylammonium bromide (CTAB) method [41].

To selectively amplify the fungal ITS2 region from the extracted DNA of the plant roots, a nested polymerase chain reaction (PCR) method was applied [33]. In the first PCR step, we amplified the entire ITS region using a fungus-specific primer (ITS1-F_KYO2 [42]) and a universal primer (ITS4 [43]) with the Ampdirect Plus (Shimadzu, Kyoto, Japan) buffer system. The ITS2 region was then amplified using a fusion primer of ITS3_KYO2 [42], which included the 454-adapter-A sequence and a 8-mer multiplex identifier (MID) tag sequence designed by Hamady *et al.* [44] to identify the source seedling (5′-CCA TCT CAT CCC TGC GTG TCT CCG ACT CAG [adapter A]–NNNNNNNN [MID]–GAT GAA GAA CGY AGY RAA [ITS3_KYO2] -3′), and a reverse fusion primer of ITS4, which included the 454-adapter-B sequence (5′-CCT ATC CCC TGT GTG CCT TGG CAG TCT CAG [adapter B]–TCC TCC GCT TAT TGA TAT GC [ITS4]-3′). The PCR products of all the 460 seedling samples were pooled in a new tube. We then purified the pooled PCR amplicons using ExoSAP-IT (GE Healthcare, Little Chalfont, Buckinghamshire, UK) and a PCR purification kit (Qiagen, Venlo, The Netherlands) before pyrosequencing using 454 GS Junior (Roche Diagnostics, Indianapolis, IN, USA). Because we did not obtain enough data in the first pyrosequencing run, we conducted an additional emulsion PCR and a pyrosequencing run. The pyrosequencing data were deposited to a public repository (DDBJ DRA: DRA000926).

### Constructing operational taxonomic units and taxonomic identification

The pyrosequencing data were processed following the method of Toju *et al.* [33]. The low-quality 3′-tails of the pyrosequencing reads obtained were trimmed based on a threshold sequence quality value of 27. The reads were then filtered by a minimum sequence length of 150 bp excluding forward primer sequences and MID tags. To obtain the molecular operational taxonomic units (OTUs), the remaining reads were assembled as follows. The reads were sorted by seedling samples using the sample-specific MID tags and assembled into contigs for each sample with a minimum sequence similarity cutoff of 97% using Assams v0.1.2012.05.24 [45] (see also [46] for detailed assembling process), which is a parallelized pipeline for implementing the assembler program Minimus [47]. This within-sample assembling helped to avoid overestimates of OTU richness [48]. Possible chimeric sequences were detected and removed using UCHIME v4.2.40 [49] with a minimum score to report a chimera of 0.1. The within-sample contigs that passed the chimera removal process were subjected to further assembling of among-sample contigs with a sequence similarity cutoff of 97% using Assams, and the among-sample contigs were analyzed in the following statistical analyses as fungal OTUs. Note that the downstream statistical results did not qualitatively differ from those obtained based on 93% and 95% cutoff similarity settings in the among-sample clustering (see Results).

We conducted a BLAST-search of the fugal OTUs using the NCBI nt database on 22 February 2013. We also attempted identification based on the lowest common ancestor (LCA) algorithm [50], of which the results were much more conservative than the BLAST top-hit matches. Specifically, the query-centric auto-*k*-nearest-neighbor (QCauto) method implemented in the program Claident v0.1.2012.05.21 [51], [52] was applied using the reference-sequence information of the “all_genus” and “all_underclass” sequence databases and the NCBI-Taxonomy information of the “all_genus” and “all_underclass” taxonomy databases (see [51] for details of those databases). The query OTU sequences are shown in Data S1.

### Binary data matrices

Based on the pyrosequencing dataset, we obtained a binary matrix that depicted the presence (1) or absence (0) of fungal OTUs in each of the 261 *Q. serrata* and 199 *Q. glauca* seedling samples (Data S2). Before obtaining the binary matrix, seedling samples with less than 20 pyrosequencing reads were excluded: 10 and nine seedling samples were excluded for *Q. serrata* and *Q. glauca*, respectively. In addition, OTUs representing less than 5% of the sample-total reads were excluded from each sample to reduce among-sample variance in *α*-diversity that resulted from variance in sequencing effort (i.e., variance in the number of sequencing reads among samples [mean  =  129.2, SD  =  69.7]). In this process, singletons and rare OTUs, which were expected to contain high proportions of pyrosequencing errors in their sequences [53], were eliminated. The eleven seedling samples that included the sequences of plants other than Fagaceae or *Quercus* spp. were also excluded from the data set: these samples were contaminated by DNAs of *Ilex*, *Prunus*, and Ericaceae plants, which commonly occurred in the study forest. Consequently, 249 *Q. serrata* and 188 *Q. glauca* seedlings were subjected to the following analyses (Data S2).

### Fungal diversity and spatial autocorrelation

Based on the presence/absence data matrix of fungal OTUs (Data S2), the diversity and spatial structure of the fungal communities on the two *Quercus* species were evaluated. To assess the species richness of fungi, species accumulation curves (Mao Tau curves) were drawn for each of the two host species using the function ‘specaccum’ in the Vegan package [54] of R (version 3.0.2 [55]). To evaluate spatial autocorrelation in fungal OTU composition within the study site, a Mantel correlogram analysis was applied to each host plant species. In the analysis, we calculated Mantel's correlation (*r*) between dissimilarity in fungal OTU composition (i.e., *β*-diversity) and Euclidean distance spanning sampling positions (999 permutations). For the calculation of *β*-diviersity, we used Raup-Crick metric [56], which could minimize statistical artifacts resulting from difference in *α*-diversity among samples (see [56]). In addition, to test the spatial autocorrelation of the occurrence of each fungal OTU within the study site, we conducted a Moran's *I* analysis [57] for each fungal OTU that occurred on 10 or more seedling samples using the R package Ape 3.0-11 [58], [59].

### Comparison of the root-associated fungal community structure between *Q. serrata* and *Q. glauca*

Prior to the statistical analysis of spatial segregation and aggregation of root-associated fungi, we examined differences in fungal community structure between *Q. serrata* and *Q. glauca* by PERMANOVA [60]. In this analysis, we measured dissimilarity in fungal OTU composition between seedling samples based on Raup–Crick *β*-diversity, and then tested for differences in the centroid of the fungal community structure of each *Quercus* species in multivariate space (9,999 permutations). The difference in the homogeneity of multivariate dispersion (the variance of *β*-diversity) between the fungal communities of the two hosts was also examined by PERMDISP [61] as implemented in Vegan.

We also examined the presence of host-specific fungal OTUs in the data set. A test using the multinomial species classification method (CLAM [62]) was performed to classify fungal OTUs preferentially associated with either host species and OTUs commonly found on both host species. A multinomial model was used to examine the statistical significance of respective fungal OTUs' preferences for host plants with a specialization threshold value of 2/3 (“supermajority” rule [62]). Because Bonferroni correction generally returns too stringent results in CLAM analysis [62], an *α* value of 0.001 was used as the threshold of statistical significance. The Vegan package of R was used in this analysis.

### Segregation and aggregation of pairs of fungi in roots

We used the Checkerboard and Togetherness scores (C score and T score [63], [64]) as indices of spatial segregation and aggregation of fungal OTUs in the seedling samples, respectively. The *C* score is defined as (*R_i_* – *S*) × (*R_j_* – *S*), where *R_i_* and *R_j_* represent the total number of occurrences of species *i* and *j*, respectively, and *S* is the number of co-occurrences [63]. The *T* score is defined as *S*(*N* + *S* – *R_i_* – *R_j_*), where *N* is the number of seedlings analyzed [64]. In the presence of antagonistic interspecific interactions or the differentiation of niches, the observed *C* score of each pair of species is expected to be greater than that obtained by randomization under a null model. However, in the presence of facilitative interspecific interactions or shared habitat requirements, the observed *T* score is expected to be greater than that obtained by randomization under a null model. In contrast, a lack of significance suggests that species co-occur randomly.

For the dataset of each host plant, we tested the significance of *C* and *T* scores with 100,000 randomizations using the Bipartite package 2.0-1 [65] of R (Test 1). Observed and randomized *C* and *T* scores were standardized to range from 0 (the possible lowest level of segregation in terms of *C* scores and the possible lowest level of aggregation in terms of *T* scores) to 1 (the possible highest level of segregation in terms of *C* scores and the possible highest level of aggregation in terms of *T* scores) [66]. In each test for *Q. serrata* or *Q. glauca* dataset, OTUs observed in five or more seedlings and OTU pairs whose sum of seedling-sample counts were 25 or more were used because the statistical significance of C and T scores was difficult to examine for fungal OTU pairs with fewer sample counts. In addition to the examination for each host plant, the randomization analysis was applied to the whole dataset including both *Q. serrata* and *Q. glauca* seedling samples: OTUs observed in 10 or more *Q. serrata* and *Q. glauca* seedlings and OTU pairs whose sum of seedling-sample counts were 50 or more were used (Test 2). False discovery rate (FDR; Benjamini-Hochberg method) control [67] was applied to each randomization analysis.

We further assessed whether or not each pair of fungal OTUs was associated with each other in different ways on different host plant species. To this end, for each pair of fungal OTUs, we calculated the difference of *C* scores between the two host plant species (i.e., *C*_serrata_ – *C*_glauca_ where *C*_serrata_ and *C*_glauca_ were standardized *C* scores on *Q. serrata* and *Q. glauca*, respectively). Likewise, the difference of *T* scores between the two host plant species (*T*_serrata_ – *T*_glauca_, where *T*_serrata_ and *T*_glauca_ were standardized *T* scores on *Q. serrata* and *Q. glauca*, respectively) was calculated for each pair of fungal OTUs. The significance of the difference of *C* or *T* scores on different host plants was tested based on 100,000 randomizations (Test 3). In this analysis, we used the fungal OTU pairs that were included in the dataset of both host plants in the Test 1. The fungal OTU pairs analyzed in the Test 1 of both host plants were used. FDR control was also applied to the analysis.

Finally, to assess whether whole community of root-associated fungi in the study site show segregated/aggregated patterns, we tested the significance of *C* and *T* scores with 100,000 randomizations. We also applied the analysis to each sub-dataset including a taxonomic or functional group of fungi (Ascomycota, Basidiomycota, and ectomycorrhizal fungal sub-datasets).

## Results

### Assembling and identification of molecular OTUs

In total, 65,150 reads were obtained by pyrosequencing (18,667 and 46,483 reads in the first and second GS Junior runs, respectively). Mean length of those reads were 348 (SD  =  69.8) bp for the first run and 362 (SD  =  55.29) bp for the second run. Only 0.175% of the reads were those of plants. The total numbers of OTUs were 1869, 1079 and 785 based on sequence cutoff similarities of 97, 95 and 93%, respectively; the numbers of singletons were 940, 414 and 270, respectively. After removing seedling samples with less than 20 pyrosequencing reads, those with ITS reads of plants other than *Quercus*, and OTUs representing less than 5% of the sample-total reads, the binary data matrix of the fungal community included 319, 274 and 242 OTUs with 97, 95 and 93%-cutoff similarities, respectively (Data S2).

**Figure 2:**
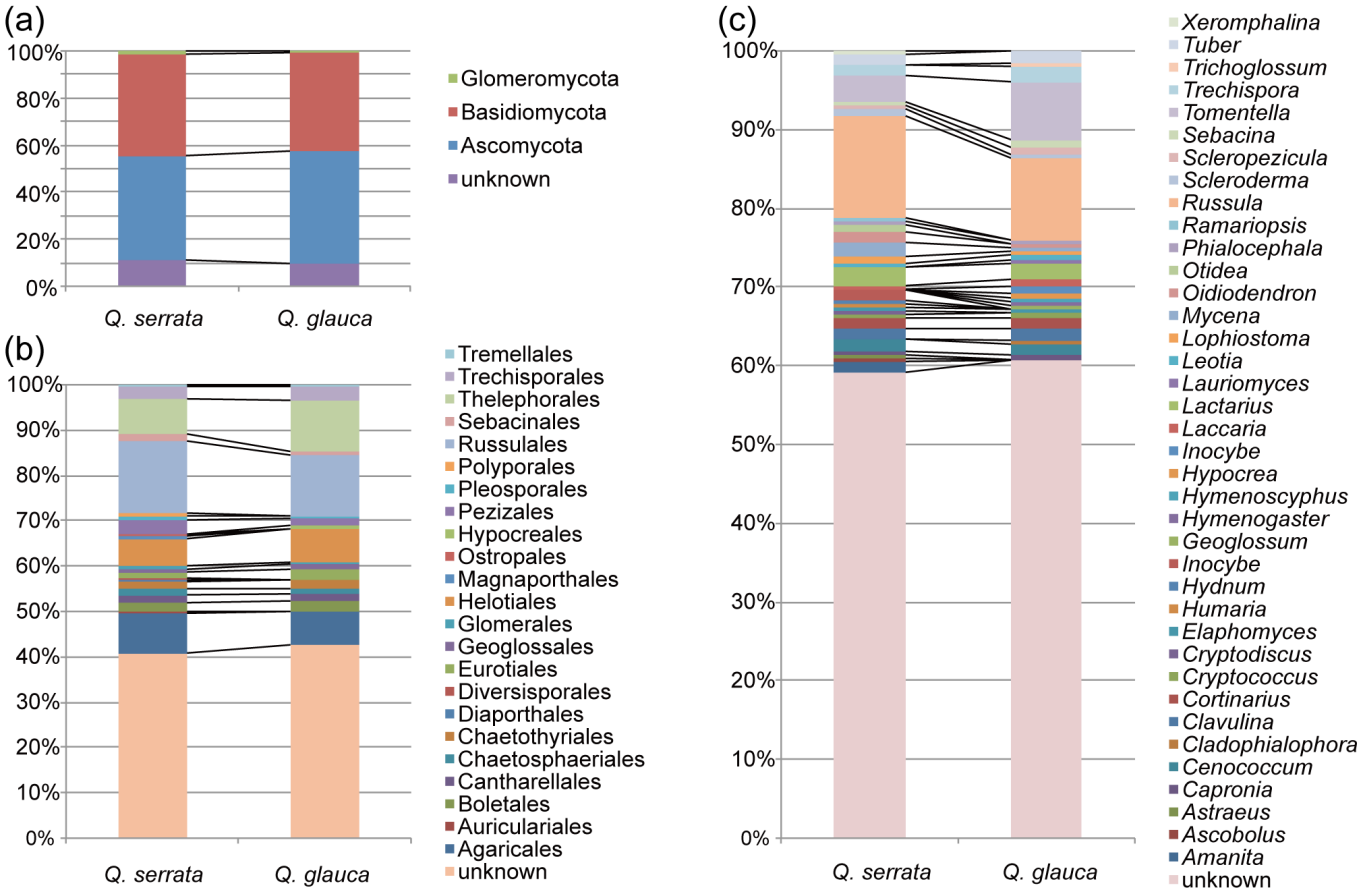
Taxonomic compositions of fungal OTUs on *Quercus serrata* and *Q*. *glauca*. (a) Phylum-level compositions of fungal OTUs. (b) Order-level compositions of fungal OTUs. (c) Genus-level compositions of fungal OTUs.

Of the 319 OTUs detected with a cutoff sequence similarity of 97%, 89.7% were identified at the phylum level, 58.9% at the order level, 50.4% at the family level, and 40.7% at the genus level (Fig. 2). From *Q. serrata* and *Q. glauca* seedlings, 94 and 86 basidiomycete, 96 and 98 ascomycete, and three and one glomeromycete OTUs were detected, respectively. Of the 319 OTUs, 34 occurred in 10 or more seedling samples. Among these 34 most common OTUs, 14 were assigned to ectomycorrhizal genera. Of the remaining 23 OTUs, three and 19 were respectively assigned to Basidiomycota and Ascomycota at the phylum level but their genera remained unidentified; the remaining one OTU could not be identified even at the phylum level by the QCauto method (Table 1). BLAST searches against the NCBI nr/nt database (Table 1) indicated that the commonly observed ascomycete OTUs were allied to genera or species that had been generally detected from living plant tissue in previous studies (i.e., possibly endophytic ascomycetes; e.g., *Catenulifera* [68], *Pezicula* [teleomorph of *Cryptosporiopsis* [69]], *Lophodermium* [70], and *Cladophialophora* [teleomorph of *Capronia* [71]), except for an OTU (OTU 121) related to soil fungi in the genus *Archaeorhizomyces* [72]. The list of commonly observed fungi included *Mycena*, *Oidiodendron*, and Glomeromycota, which are known as saprobes or ericoid/arbuscular mycorrhizal fungi (Table S1). We found no inconsistency between the QCauto-based and BLAST-based identification results, although results by the QCauto method were more conservative than those of BLAST (Table 1). Taxonomic diversity of OTUs were qualitatively similar among identification results with different sequence-similarity cutoffs (see Tables S1 and S2 for results at 93% and 95% cutoffs).

**Table 1:** List of molecular OTUs occurring in 10 or more seedlings.

ID	CLAM test	Moran's *I*	Number of occurrences		BLAST top-hit result						Taxonomic assignment using the QCauto method	Type
	Preffered host		*Q. serrata*	*Q. glauca*	Description	TS	QC	*E* value	Identity	Accession		
757	Both	0.0215	106	56	*Catenulifera luxurians*	462	91%	6E-127	293/314	GU727560	phylum: Ascomycota; class: Leotiomycetes	
167	Both	0.0053	40	31	*Cenococcum geophilum*	555	91%	1E-154	309/313	JQ711949	class: Dothideomycetes; genus: *Cenococcum*	EcM
115	Both	0.0109	36	20	*Catenulifera brevicollaris*	483	91%	5E-133	299/317	GU727561	subkindom: Dikarya; phylum: Ascomycota	
329	Both	0.0192	31	16	*Pezicula sp.*	499	91%	5E-138	299/313	AB731133	order: Helotiales; family: Dermateaceae	
1845	Both	0.0144	27	20	*Cryptosporiopsis* sp.	375	91%	8E-101	275/310	JN601680	class: Leotiomycetes; order: Helotiales	
387	Both	−0.0127	29	16	*Leptodontidium* sp.	486	80%	4E-134	270/273	DQ069033	class: Leotiomycetes; order: Helotiales	
193	Both	0.0149	26	16	*Russula cerolens*	675	93%	0E+00	384/393	JN681168	family: Russulaceae; genus: *Russula*	EcM
199	Both	0.0527*	18	20	*Cryptosporiopsis* sp.	379	91%	6E-102	277/312	JN601680	subkindom: Dikarya; phylum: Ascomycota	
203	Both	−0.0169	19	18	*Lophodermium jiangnanense*	267	92%	5E-68	265/321	GU138714	phylum: Ascomycota; class: Leotiomycetes	
205	Both	0.0201	12	18	*Arcangeliella camphorata*	678	93%	0E+00	408/426	EU644700	family: Russulaceae; genus: *Lactarius*	EcM
331	Both	0.0073	19	9	*Cladophialophora carrionii*	497	93%	2E-137	326/352	HM803232	order: Chaetothyriales; family: Herpotrichiellaceae	
121	Both	0.0635*	17	9	*Archaeorhizomyces finlayi*	159	90%	9E-36	255/291	JQ912673	subkindom: Dikarya; phylum: Ascomycota	
1089	*Q. serrata*	0.0310	25	0	*Lactarius quietus*	767	89%	0E+00	419/421	JF273529	species: *Lactarius quietus*	EcM
211	Both	0.0903*	11	12	*Thelephora terrestris*	647	92%	0E+00	374/386	JX030236	family: Thelephoraceae; genus: *Thelephora*	EcM
169	Both	0.0730*	14	5	*Tomentella* sp.	689	90%	0E+00	375/376	JF2735461	family: Thelephoraceae; genus: *Tomentella*	
823	Both	0.0174	14	5	*Tomentella* sp.	593	80%	2E-166	327/330	HE814132	order: Thelephorales; family: Thelephoraceae	
867	Both	0.0056	17	2	*Rhizoscyphus ericae*	473	93%	3E-130	294/312	JQ711893	subkindom: Dikarya; phylum: Ascomycota	
425	Both	0.0567*	7	11	*Clavulina* sp.	710	90%	0E+00	384/384	JF273519	family: Clavulinaceae; genus: *Clavulina*	EcM
185		0.0137	15	1	*Russula japonica*	577	87%	2E-161	344/358	AB509603	family: Russulaceae; genus: *Russula*	EcM
375	Both	−0.0035	9	7	*Absconditella lignicola*	241	89%	3E-60	219/260	FJ904669	kingdom: Fungi; subkingdom: Dikarya	
411		−0.0016	7	7	*Tomentella* sp.	612	93%	6E-172	369/387	FM955848	order: Thelephorales; family: Thelephoraceae	
527		0.0057	4	10	*Graddonia coracina*	353	91%	4E-94	277/317	JQ256423	subkindom: Dikarya; phylum: Ascomycota	
207		−0.0006	11	1	*Cryptosporiopsis* sp.	374	93%	3E-100	276/312	JN601680	subkindom: Dikarya; phylum: Ascomycota	
393		0.0482*	5	7	*Penicillium* sp.	272	92%	1E-69	286/348	FJ379804	order: Eurotiales; family: Trichocomaceae	
517		0.0120	10	2	*Cenococcum geophilum*	540	91%	3E-150	305/311	HM189732	class: Dothideomycetes; genus: *Cenococcum*	EcM
1135		0.2397*	5	7	*Russula* sp.	712	85%	0E+00	388/389	HE814200	family: Russulaceae; genus: *Russula*	EcM
157		0.0058	10	1	*Cortinarius* sp.	492	91%	8E-136	295/309	JQ272415	subkindom: Dikarya; phylum: Ascomycota	
349		−0.0122	8	3	*Russula vesca*	549	92%	5E-153	365/394	AY606965	family: Russulaceae; genus: *Russula*	EcM
423		−0.0116	5	6	*Brevicellicium olivascens*	315	96%	2E-82	315/382	JN649327	class: Agaricomycetes; order: Trechisporales	
1157		0.0019	8	3	*Russula* sp.	676	88%	0E+00	366/366	AB531451	family: Russulaceae; genus: *Russula*	EcM
113		−0.0025	3	7	*Cenococcum geophilum*	520	91%	4E-144	306/317	EU427331	class: Dothideomycetes; genus: *Cenococcum*	EcM
153		0.0181	6	4	*Elaphomyces decipiens*	505	100%	1E-139	327/352	EU837229	family: Elaphomycetaceae; genus: *Elaphomyces*	EcM
195		0.0289	8	2	*Parmelia* sp.	436	88%	4E-119	274/293	HQ671309	phylum: Ascomycota; class: Dothideomycetes	
623		0.0126	3	7	*Sphaerosporella* sp.	313	94%	7E-82	248/285	JQ711781	subkindom: Dikarya; phylum: Ascomycota	

Legend: Columns indicate the results of CLAM tests and Moran's *I* analyses, the number of occurrences in each *Q. serrata* (*n*  =  249) and *Q glauca* (*n*  =  188), taxonomic identification results based on BLAST searches and the QCauto method, and the putative fungal functional type. In the column of BLAST top-hit result, total Blast score (TS), query coverage (QC), and identity (number of identical sites/number of the sites aligned to those of the BLAST top-hit sequence) are shown.

* *P* < 0.05 after FDR control.

### Fungal diversity and spatial autocorrelation

Of the 319 OTUs detected with a cutoff sequence similarity of 97%, 103 occurred on both *Q. serrata* and *Q. glauca*, while 115 and 101 OTUs occurred only on either *Q. serrata* or *Q. glauca*, respectively (Fig. 1a). Species accumulation curves did not reach a plateau for either host-plant species (Fig. 1b). The average number of OTUs per seedling was almost similar between the two host plant species but it was statistically higher on *Q. serrata* than on *Q. glauca* (3.51 and 3.20 on *Q. serrata* and *Q. glauca*, respectively; *t*  =  2.12, df  =  414.377, *P* < 0.05).

**Figure 3:**
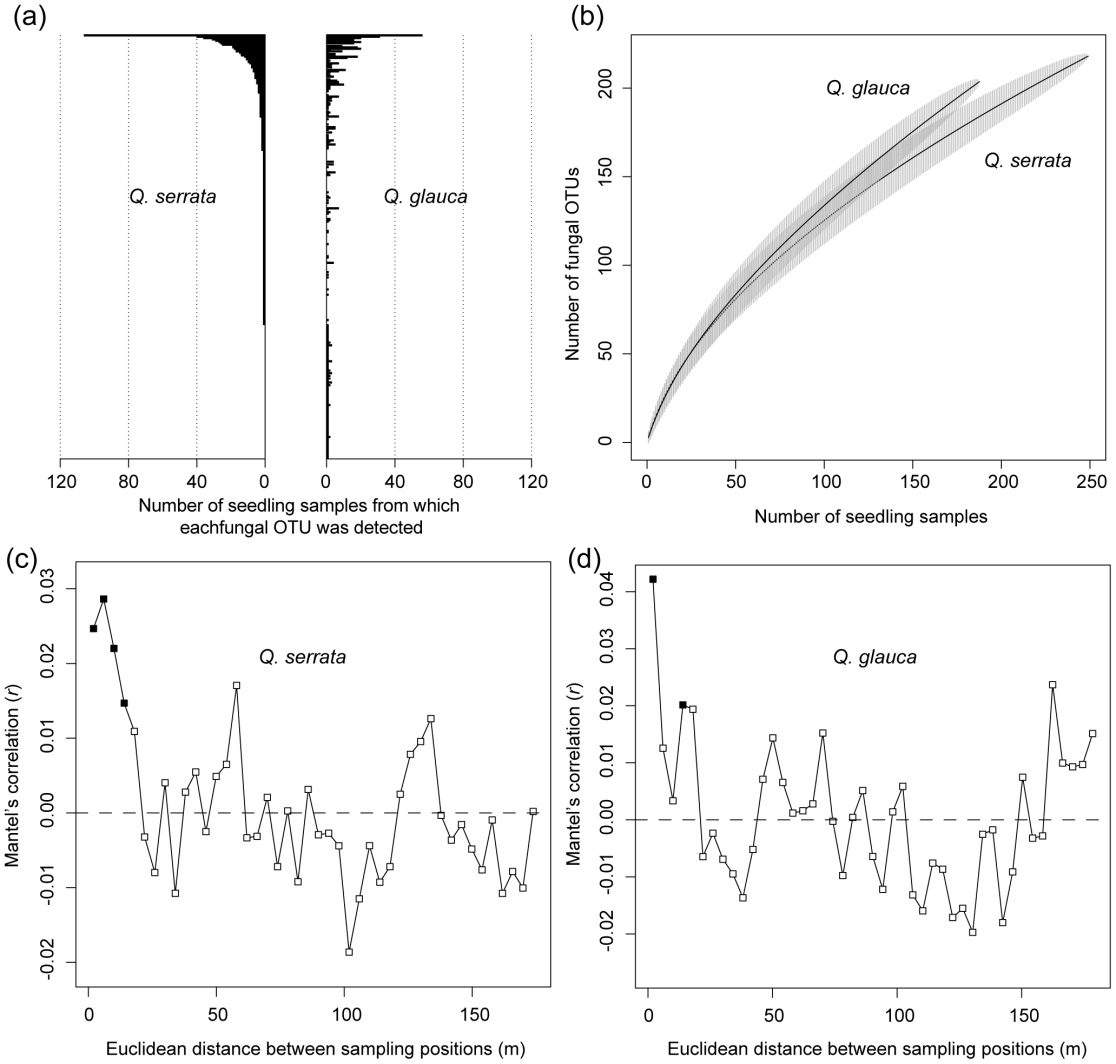
Root-associated fungal diversity on *Quercus serrata* and *Q*. *glauca*. (a) Number of seedling samples from which each fungal OTU was detected. (b) Accumulation curves of fungal OTUs against the number of *Q. serrata* or *Q. glauca* seedlings. The solid line and the gray area denote the expected mean OTU richness and its standard deviation, respectively. (c, d) Spatial autocorrelation analysis of fungal OTU compositions. A Mantel correlogram analysis was conducted to evaluate the extent of the spatial autocorrelation of root-associated fungal OTU compositions. Mantel's *r* statistic representing the correlation between dissimilarity in fungal OTU compositions (Raup-Crick *β*-diversity) and Euclidean distance spanning sampling positions is shown for each distance class. Filled symbols represent significantly positive spatial autocorrelation. (c) Analysis on *Q. serrata* with intervals of ca. 2 m. (d) Analysis on *Q. glauca* with intervals of ca. 2 m.

A Mantel correlogram analysis indicated that the fungal OTU composition of the examined seedling samples was spatially auto-correlated at a very small spatial scale within the study site: i.e., the scale of the autocorrelation was < ca. 8 m for *Q. serrata* seedlings and < ca. 2 m for *Q. glauca* seedlings (Fig. 3c, d). In a Moran's *I* analysis, significant spatial autocorrelation within the study site was observed for only seven of the 34 common fungal OTUs examined (Table 1).

### Comparison of root-associated fungal community structures between *Q. serrata* and *Q. glauca*

Although PERMANOVA analysis indicated a significant difference in community structures between the two host species (*F*  =  4.79, *P*  =  0.0002), the *R* ^2^ value was very low (*R* ^2^  =  0.0109), suggesting that the effects of host plant identity on fungal OTU compositions were small in the present dataset. In fact, the community compositions of root-associated fungi on *Q. serrata* and *Q. glauca* were largely similar to each other (Fig. 2). The PERMDISP analysis showed significant differences in the among-sample *β*-diversity (Raup-Crick) of fungal communities between the two host-plant species. The *β*-diversity of the fungal communities was greater on *Q. glauca* than on *Q. serrata* (average distance between each fungal community and the centroid of fungal community were 0.5765 for *Q. serrata* and 0.6283 for *Q. glauca*, *F*  =  12.61, *P*  =  0.0004). Consistent results were obtained in the analyses based on 95% and 93% cutoff sequence similarities: for simplicity, results at 97% cutoff sequence similarity are shown in the following statistical analyses.

The CLAM test revealed the presence of 18 OTUs commonly associated with both *Quercus* species and an OTU exclusively associated with *Q. serrata*, although many OTUs were too rare to be assigned host preference (Table 1). The OTU that exclusively occurred on *Q. serrata* (OTU 1089) showed 99% sequence similarity to *Lactarius quietus* (JF273529). The list of fungi commonly associated with both plant species included fungal OTUs related to various ectomycorrhizal fungi, e.g., *Cenococcum geophilum* (OTU 167), *Russula cerolens* (OTU 193), and *Thelephora terrestris* (OTU 211), and those related to possibly endophytic fungi in the orders Chaetothyriales and Helotiales, e.g., *Catenulifera luxurians* (OTU 757) and *Pezicula* sp. (OTU 329).

### Segregation and aggregation of pairs of fungi in roots

When the seedling samples of the two host plants were analyzed separately (Test 1), no pair of fungal OTUs showed significant *C* or *T* scores after adjusting *P* values based on FDR control. However, many pairs of fungal OTUs displayed low (< 0.005) *P* values without FDR control (Table 2). In the *C* score analysis, an ectomycorrhizal ascomycete in the genus *Cenococcum* (OTU 167) and a possibly endophytic ascomycete (OTU 757) displayed segregated distribution on *Q. serrata* (Table 2). In the *T* score analysis, eight pairs of fungal OTUs displayed aggregated patterns with low (< 0.005) *P* values on either of the two host plants. Among the eight pairs, six were pairs of an ectomycorrhizal basidiomycete (*Russula* [OTU 185, 193, 509, or 1135] or *Lactarius* [OTU 205]) and a possibly endophytic ascomycete fungus (OTU 115 or 331; Table 2). The remaining two pairs were those of possibly endophytic ascomycetes (Table 2).

**Table 2:** List of fungal OTU pairs that displayed segregated or aggregated patterns when each host plant species was analyzed independently (Test 1).

Pair of OTUs				*C* or *T* score	*P*	FDR§
IDs (Taxonomic information)†						
*Q. serrata*						
Segregation (*C* score analysis)						
757	(class: Leotiomycetes)	167	(genus: *Cenococcum*)*	0.679	0.0042	1.000
Aggregation (*T* score analysis)						
509	(genus: *Russula*)*	115	(phylum: Ascomycota)	0.072	0.0010	0.300
193	(genus: *Russula*)*	115	(phylum: Ascomycota)	0.133	0.0016	0.300
331	(family: Herpotrichiellaceae)	185	(genus: *Russula*)*	0.075	0.0034	0.409
*Q. glauca*						
Aggregation (*T* score analysis)						
331	(family: Herpotrichiellaceae)	205	(genus: *Lactarius*)*	0.115	0.0007	0.124
375	(subkingdom: Dikarya)	203	(class: Leotiomycetes)	0.093	0.0024	0.154
199	(phylum: Ascomycota)	1845	(order: Helotiales)	0.150	0.0030	0.154
115	(phylum: Ascomycota)	1135	(genus: *Russula*)*	0.092	0.0038	0.154
193	(genus: *Russula*)*	115	(phylum: Ascomycota)	0.131	0.0043	0.154

Legend: The significance of *C* or *T* scores was examined based on a randomization test for each pair of fungal OTUs (100,000 permutations).

† For each fungal OTU, taxonomic information based on the QCauto method is shown. Asterisks indicate possibly ectomycorrhizal OTUs.

§ Adjusted *P* values (FDR control).

In the analysis in which the datasets of the two host plants were combined (Test 2), three pairs of fungal OTUs displayed statistically significant aggregated patterns even after FDR control (Table 3). Among the three pairs, two were those of an ectomycorrhizal basidiomycete (*Russula* [OTU 193] or *Lactarius* [OTU 205]) and a possibly endophytic ascomycete (OTUs 115 or 331). The remaining pair was that of possibly endophytic ascomycetes (OTUs 199 and 1845). Note that such aggregation of pairs of ectomycorrhizal and endophytic fungi or those of endophytic fungi was also observed in the datasets based on 93% or 95% cutoff similarities (Table S2). In addition to the abovementioned pairs, two pairs of an ectomycorrhizal basidiomycete (*Russula* [OTU 1135] or *Lactarius* [OTU 1089]) and a possibly edophytic ascomycete (OTU 115 or 331) and a pair of possibly root-endophytic ascomycete fungi displayed aggregated patterns with low (< 0.005) *P* values (Table 3). Likewise, segregated patterns with low *P* values were observed for a pair of an ectomycorrhizal ascomycete (*Cenococcum* [OTU 167]) and a possibly endophytic ascomycete (OTU 757), although the patterns were non-significant after FDR control (Table 3).

**Table 3:** List of fungal OTU pairs that displayed segregated or aggregated patterns when the seedling samples of the two *Quercus* species were analyzed simultaneously (Test 2).

Pair of OTUs				*C* or *T* score	*P*	FDR§
IDs (Taxonomic information)†						
Segregation (*C* score analysis)						
757	(class: Leotiomycetes)	167	(genus: *Cenococcum*)*	0.698	0.0014	0.359
Aggregation (*T* score analysis)						
193	(genus: *Russula*)*	115	(phylum: Ascomycota)	0.131	0.0000	0.003
199	(phylum: Ascomycota)	1845	(order: Helotiales)	0.101	0.0004	0.032
331	(family: Herpotrichiellaceae)	205	(genus: *Lactarius*)*	0.072	0.0004	0.032
375	(subkingdom: Dikarya)	203	(class: Leotiomycetes)	0.054	0.0014	0.084
115	(phylum: Ascomycota)	1135	(genus: *Russula*)*	0.052	0.0021	0.104
331	(family: Herpotrichiellaceae)	1089	(species: *Lactarius quietus*)*	0.054	0.0043	0.180

Legend: The significance of *C* or *T* scores was examined based on a randomization test for each pair of fungal OTUs (100,000 permutations).

† For each fungal OTU, taxonomic information based on the QCauto method is shown. Asterisks indicate possibly ectomycorrhizal OTUs.

§ Adjusted *P* values (FDR control).

When the effects of host plants on the segregation/aggregation of fungal OTUs were examined (Test 3), no pair of fungal OTUs showed significant difference of *C* or *T* scores between *Q. serrata* and *Q. glauca* after FDR control; even without FDR control, no fungal pair showed low (< 0.005) *P* values (Table S3). These results indicate that the identity of host plant species did not affect the segregation/aggregation patters of root-associated fungi in the present dataset.

In the community-scale analysis of *C* scores (Table 4), significantly segregated patterns were observed within the entire community of the observed fungi and within the basidiomycete and ectomycorrhizal fugal sub-communities, while no sign of segregation was observed within the ascomycete sub-community. As expected by the *C* score analysis, observed values of togetherness (*T*) were very low (Table 5).

**Table 4:** Analysis of segregated patterns (i.e., *C* score analysis) within each taxonomic or functional group of fungi.

Subset of community	*Q. serrata*			*Q. glauca*			Both host species		
	*C* score	*P* (FDR)	SES§	*C* score	*P* (FDR)	SES§	*C* score	*P* (FDR)	SES§
All	0.9741	**0.0091**	2.37	0.9757	0.0544	1.69	0.9823	**0.0127**	2.27
Ascomycota	0.9609	0.2553	0.69	0.9622	0.9157	−1.42	0.9718	0.6499	−0.37
Basidiomycota	0.9849	**0.0004**	3.25	0.9859	**0.0080**	2.67	0.9895	**0.0008**	3.54
EcM	0.9814	**0.0018**	2.84	0.9791	0.0544	1.66	0.9860	**0.0046**	2.68

Legend: The significance of *C* scores was examined based on a randomization test (100,000 permutations). FDR control was applied to each (sub-)dataset: significant *P* values are indicated in bold.

§ SES indicates standard effect size.

**Table 5:** Analysis of aggregated patterns (i.e., *T* score analysis) within each taxonomic or functional group of fungi.

Subset of community	*Q. serrata*			*Q. glauca*			Both host species		
	*T* score	*P*	SES§	*T* score	*P*	SES§	*T* score	*P*	SES§
All	0.0004	0.9962	−2.51	0.0004	0.9975	−2.45	0.0004	1.0000	−3.43
Ascomycota	0.0007	0.4216	0.17	0.0008	0.1083	1.26	0.0008	0.1720	0.96
Basidiomycota	0.0005	1.0000	−4.15	0.0004	1.0000	−3.64	0.0005	1.0000	−5.54
EcM fungi	0.0005	1.0000	−4.06	0.0004	0.9979	−2.56	0.0005	1.0000	−4.95

Legend: The significance of *T* scores was examined based on a randomization test (100,000 permutations). FDR control was applied to each (sub-)dataset.

§ SES indicates standard effect size.

## Discussion

In the present study, the sign of segregated or aggregated patterns was detected for a small number of fungal OTU pairs. Although the results may reflect the rarity of competitive or facilitative interactions between root-associated fungi in the study forest, there are potential statistical issues that may have hampered the detection of significant patterns. To make clear the potential pitfalls in pyrosequencing-based high-throughput analyses of segregated/aggregated distributions of fungi, we start with discussing problems related to multiple comparisons and the power of randomization tests.

When the checkerboard (*C*) and togetherness (*T*) scores of pairs of fungal OTUs were examined on each of the two host plant species (Test 1), no fungal pair showed statistically significant segregation nor aggregation patterns after FDR control (Table 2). Given that more than 179 pairs of fungal OTUs were examined in our pyrosequencing-based analysis, the application of the multiple comparison method might make the results prone to type II errors [73]. In the studies of segregation/aggregation patterns of root-associated fungi, such a statistical issue has been generally underappreciated. For example, a previous study discussed spatial segregation or aggregation of fungal species without any multiple-comparison adjustment of *P*-values [26], thereby making their results prone to type I errors (false positives) rather than type II errors (false negatives). Therefore, by applying multiple comparison methods to the datasets of the previous studies, one may be able to screen for pairs of fungi with strong sign of segregated/aggregated patterns.

In addition to the possible effects of multiple comparison methods, problems related to sample size could affect the results of randomization test. Intriguingly, when the samples of the two host species were pooled in the *C* and *T* score analysis (Test 2), three pairs of fungal OTUs showed statistically significant aggregation even after FDR control (Table 3). Given that the identity of host plants was not associated with the segregation/aggregation patterns of root-associated fungi in the current dataset (Test 3; Table S3), the fact that significant segregation/aggregation was observed in the simultaneous analysis of *Q. serrata* and *Q. glauca* seedlings (Test 2) but not in the independent analysis of them (Test 1) might be attributed to the larger number of samples in Test 2 than in Test 1. Importantly, when the sample counts of examined fungi (i.e., the number of seedling samples in which respective fungi occurred) are small, *C* or *T* scores of randomized data matrices frequently take the minimum (0) or maximum (1) values and hence deviate from normal distribution (Fig. S1), possibly reducing the power of the randomization tests. Therefore, in the present study, sample counts of fungi would have been insufficient to detect segregation/aggregation patterns independently on each host plant (Test 1), despite the intensive sampling of seedlings in the forest (249 *Q. serrata* and 188 *Q. glauca* seeding samples). Overall, the comparison of the results between Test 1 and Test 2 suggests that fungal pairs with strong segregation/aggregation signs become detectable by increasing sample size (and thereby sample counts; Fig. S1).

Although the abovementioned statistical issues should be treated with caution, the present results have important implications for competitive or facilitative interactions between root-associated fungi. Albeit non-significant when FDR control was applied, a segregated pattern with a low *P* value (< 0.005) was observed between an ectomycorrhizal fungus in the ascomycete genus *Cenococcum* and a possibly endophytic ascomycete fungus (Tables 2 and 3). This result is intriguing, given that most previous studies investigated the potential competitive interactions *within* ectomycorrhizal or root-endophytic fungal communities [19], [20], [26], [27] (see also [30], Tables 4 and 5) and did not assume that ectomycorrhizal fungi could affect the spatial distribution of endophytic (or other types of non-ectomycorrhizal) fungi and vice versa. However, as suggested by our present results, competition for space or resources within root systems could occur not only between fungi with similar ecological or physiological properties but also between fungi in different functional groups. Similar phenomena were reported in a study of root-associated fungi on an ericaceous plant [36]. Combined with the findings of the previous study, our present results suggest that simultaneous analysis of multiple functional groups would give novel insights into the community dynamics of root-associated fungi.

In addition to spatial segregation, aggregated patterns of pairs of fungi were observed in this study (Tables 2 and 3). These pairs included those of an ectomycorrhizal fungus in the genus *Russula* or *Lactarius* and a possibly-endophytic ascomycete fungus (Tables 2 and 3). Such co-occurrence of ectomycorrhizal and possibly endophytic fungi in single root system has been inferred from other lines of evidence [30]-[33], suggesting that specific pairs of fungi in different functional groups can interact with each other, potentially in facilitative or mutualistic ways. However, aggregated patterns can be observed between fungal species with similar physiological requirements for root environments, and the observed coexistence of possibly endophytic ascomycete fungi (Tables 2 and 3) may be attributed to shared preferences for habitats. Given that we focused on the segregated/aggregated patterns of fungi at the scale of host seedling individuals, potential vertical heterogeneity of microenvironments (e.g., soil nutrient availability) and the resultant partitioning of niches within root systems might have produced the observed aggregated patterns. Therefore, further studies are required to confirm whether the observed aggregated patterns reflect actual fungus–fungus ecological interactions in root systems. For example, experimental-inoculation methods will help to examine what kinds of mechanisms are responsible for the co-occurrence of those specific pairs of fungi.

Despite the proposition that fungus–fungus competitive or facilitative interactions could vary depending on host plant species, no significant difference in the levels of spatial segregation or aggregation was observed between *Q. serrata* and *Q. glauca* samples. The lack of host effects on the segregated or aggregated patterns may be partly attributed to the phylogenetic closeness of the two host species. Given that the root-associated fungal community composition was highly similar between the congeneric plant species (Fig. 2; cf. [74]), the environmental conditions experienced by root-associated fungi might be almost identical between the two host species. Hence, the use of congeneric host plant species in the present study may have precluded the detection of hosts' effects on fungus–fungus interactions. Therefore, the present results do not necessarily mean that host plants generally have no impact on the nature or strength of fungus–fungus interactions in roots: comparative analyses of *C* or *T* scores on phylogenetically-distant host plant species are awaited to further discuss the potential effects of host plants on the assembly processes of root-associated fungi.

As shown in this study, pyrosequencing technologies allow high-throughput profiling of root-associated fungal communities in hundreds (or more) of host individuals and will offer a breakthrough in the investigation of fungus–fungus interactions in roots. In addition, sequence-based taxonomic assignment (identification) potentially allows us to assess intraspecific genotypic diversity [75], providing further opportunities to infer fungus–fungus ecological/evolutionary interactions. Meanwhile, this pyrosequencing approach is based entirely on observational data on the co-occurrence of fungal OTUs in roots, and hence, it only provides insights into the potential consequences of competitive or facilitative interactions between root-associated fungal species/taxa. Therefore, complementary experimental studies are important to reveal the nature of fungus–fungus ecological interactions. For example, some experimental approaches have separated the mechanisms and consequences of interspecific interactions between fungal species [23], [24], [30] and others have quantitatively evaluated the effect of fungus–fungus interactions on host's performance [75], [76]. In combination with such experimental studies, the pyrosequencing-based high-throughput profiling of fungal communities will contribute to our understanding of the assembly processes of ecologically diverse root-associated fungi.

## Supporting Information

Figure S1 **Histograms of** ***C*** **and** ***T*** **scores that were obtained from the randomization of simulated data.** In each combination of sample size (*N*) and sample counts of OTUs (*R_i_* and *R_j_*), the histogram of *C* or *T* scores of randomized data matrix were obtained. *C* and *T* scores tend to take the maximum (1) and minimum (0) values, respectively, when sample sizes (*N*) or sample counts of fungal OTUs (*R_i_* and *R_j_*) are small. Likewise, when *R_i_* and *R_j_* are much smaller than *N*, *C* and *T* scores tend to take the extreme values. Thus, all of sample size, sample counts of fungal OTUs, and the balance between them should be carefully inspected when screening pairs of fungal OTUs prior to randomization tests of *C* or *T* scores
(TIF)

Table S1 Results of taxonomic assignment using QCauto method.
(XLSX)

Table S2 Tables with a list of fungal OTU (defined with cutoff similarities of 93 and 95%) pairs that displayed segregated or aggregated patterns.
(XLSX)

Table S3 Tables with a list of fungal OTU (defined with cutoff similarity of 97%) pairs that displayed segregated or aggregated patterns.
(XLSX)

Data S1 **Consensus ITS sequence of each molecular OTU in Fasta format.**
(TXT)

Data S2 **Binary community data matrix.**
(CSV)
