# Supplementary figures and images for: Spatial Segregation and Aggregation of Ectomycorrhizal and Root-Endophytic Fungi in the Seedlings of Two Quercus Species
Source: PLoS One. 2014 May 6;9(5):e96363. doi: 10.1371/journal.pone.0096363 (PMC4011744; doi:10.1371/journal.pone.0096363)

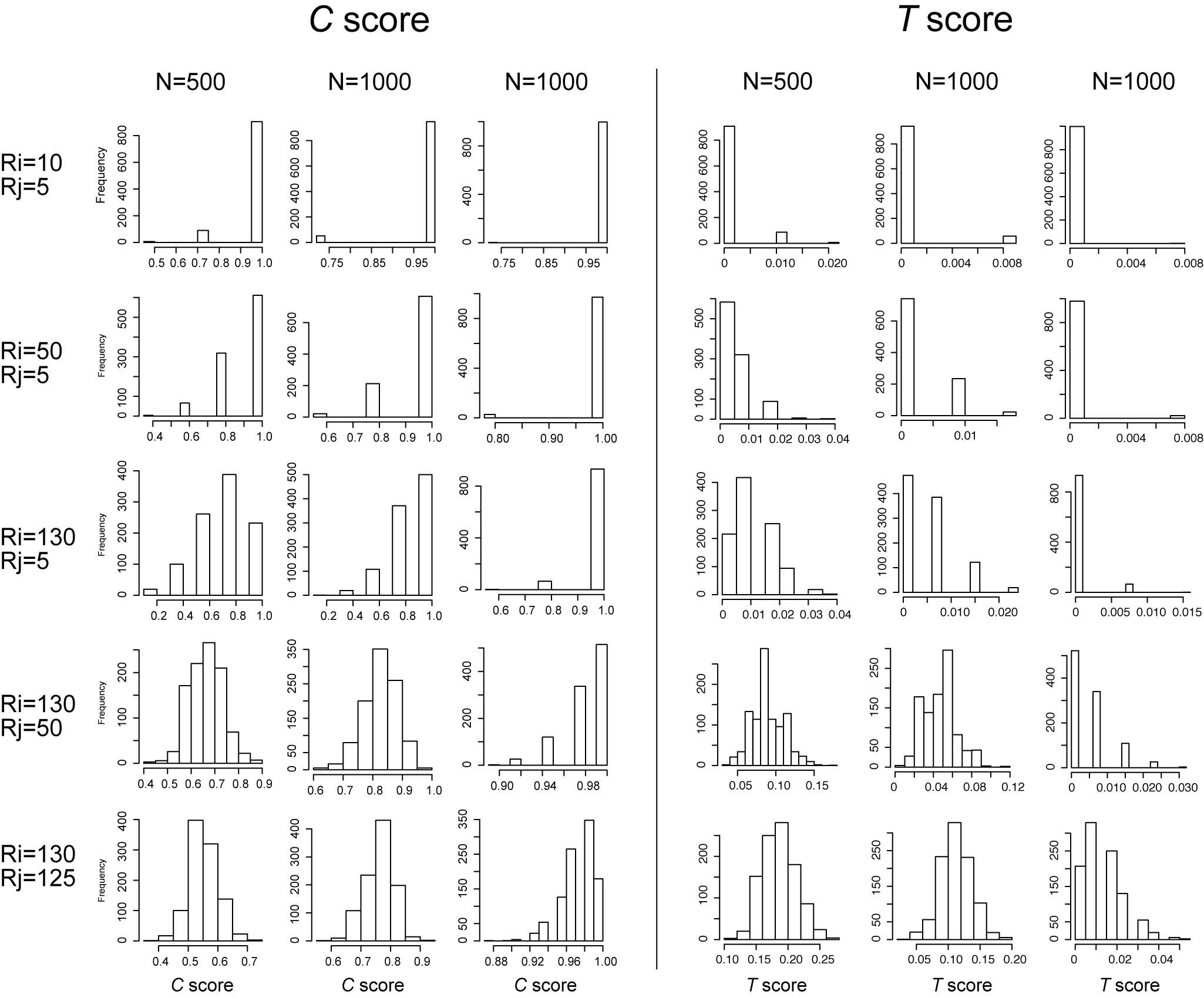

Supplement: Figure S1 — Histograms of C and T scores that were obtained from the randomization of simulated data. In each combination of sample size (N) and sample counts of OTUs (Ri and Rj), the histogram of C or T scores of randomized data matrix were obtained. C and T scores tend to take the maximum (1) and minimum (0) values, respectively, when sample sizes (N) or sample counts of fungal OTUs (Ri and Rj) are small. Likewise, when Ri and Rj are much smaller than N, C and T scores tend to take the extreme values. Thus, all of sample size, sample counts of fungal OTUs, and the balance between them should be carefully inspected when screening pairs of fungal OTUs prior to randomization tests of C or T scores (TIF) [file pone.0096363.s001.tif]
